# Non-Susceptibility Gene Variants in Head and Neck Paragangliomas

**DOI:** 10.3390/ijms252312762

**Published:** 2024-11-27

**Authors:** Anastasiya V. Snezhkina, Vladislav S. Pavlov, George S. Krasnov, Dmitry V. Kalinin, Elena A. Pudova, Olga V. Stolbovskaya, Anastasiya V. Dunshina, Maria S. Fedorova, Anna V. Kudryavtseva

**Affiliations:** 1 Engelhardt Institute of Molecular Biology, Russian Academy of Sciences, 119991 Moscow, Russia; 2 Vishnevsky Institute of Surgery, Ministry of Health of the Russian Federation, 117997 Moscow, Russia; 3 Department of Human Anatomy, Ulyanovsk State University, 432017 Ulyanovsk, Russia; 4 Ulyanovsk Regional Clinical Oncological Dispensary, 432068 Ulyanovsk, Russia

**Keywords:** head and neck paragangliomas, genetic screening, mutations, gene network

## Abstract

Head and neck paragangliomas (HNPGLs) are rare neoplasms that, along with pheochromocytomas and extra-adrenal paragangliomas, are associated with inherited mutations in at least 12 susceptibility genes in approximately 40% of cases. However, due to the rarity of HNPGLs, only a series of small-scale studies and individual cases have reported mutations in additional genes that may be involved in tumorigenesis. Consequently, numerous disease-causing mutations and genes responsible for the pathogenesis of HNPGLs remain poorly investigated. The aim of this study was to gain a deeper understanding of the genetic basis of HNPGLs by focusing on variants in genes that were not previously identified as well-known drivers. A whole-exome data analysis was conducted on a representative set of 152 HNPGLs. In 30% of the tumors examined, 53 potentially deleterious variants were identified in 36 different genes. The analysis identified pathogenic or likely pathogenic variants in the *ARNT*, *IDH2*, *L2HGDH*, *MYH3*, *PIK3CA*, and *TERT* genes. A functional network analysis of the mutated genes revealed numerous associations and a list of metabolic pathways (e.g., the TCA cycle, carbon metabolism, pyruvate metabolism, etc.) and signaling pathways (e.g., HIF1, PI3K-Akt, FoxO, AMPK, MAPK, etc.) that may play an important role in the development of HNPGLs. The identified range of genetic alterations affecting multiple genes and, potentially, influencing diverse cellular pathways provides an enhanced molecular genetic characterization of HNPGLs.

## 1. Introduction

Paragangliomas (PGLs) and pheochromocytomas (PCCs), collectively designated as PPGLs, are rare neoplasms that originate from neural crest-derived paraganglion cells and are associated with the autonomic nervous system [1]. The term “pheochromocytoma” is used to refer to tumors that develop from an adrenal sympathetic paraganglion, whereas tumors that arise from the extra-adrenal paraganglia are referred to as paragangliomas. The latter can be situated in regions of the parasympathetic nervous system in the head and neck. Approximately 20% of PPGLs are head and neck paragangliomas (HNPGLs), with an incidence rate of one case per 30,000–100,000 individuals annually [2]. HNPGLs are classified according to their anatomical location, which most commonly includes the carotid artery bifurcation (carotid paragangliomas, CPGLs), paraganglion around the vagus nerve (vagal paragangliomas, VPGLs), and the middle ear (middle ear paragangliomas, MEPGLs). It is possible for HNPGLs to develop as multifocal tumors with bilateral tumor distribution and together with PPGLs of different sites, as well as with other tumors in syndromic lesions [2]. A diagnosis of HNPGLs requires the utilization of instrumental diagnostic techniques, including computed tomography, magnetic resonance imaging, digital subtraction angiography, and color-coded Doppler sonography [3]. Molecular diagnostics have yet to be developed. For the majority of patients with HNPGLs, surgical intervention represents the primary course of treatment. Nevertheless, this approach presents a significant challenge due to the complex tumor location in the vicinity of major blood vessels and cranial nerves [4]. In recent years, there has been a growing interest in the investigation of genetic changes and specific biomolecules in PPGLs in the context of the development of personalized molecular targeted therapy [5].

The hereditary background of PPGLs is estimated to be approximately 40% [6]. At least 12 well-known genes have been linked to hereditary tumors, including *SDHA*, *SDHB*, *SDHC*, *SDHD*, *SDHAF2*, *MAX*, *FH*, *NF1*, *RET*, *TMEM127*, *VHL*, and *SLC25A11* [7,8,9]. Additionally, germline mutations were also identified in *EGLN1*, *EGLN2*, *MDH2*, *KIF1B*, *MEN1*, *MET*, *MDH2*, *GOT2*, *DLST*, *IDH3B*, *KIF1B*, *MERTK*, *KMT2B*, *PDK1*, *REXO2*, and other genes with a low prevalence [10,11,12,13,14,15,16,17,18]. A number of susceptibility genes, including *SDHx*, *RET*, *VHL*, *NF1*, and *MAX*, have been identified as harboring somatic mutations. Additionally, a wide range of other genes have been found to carry somatic mutations, including *HIF2A/EPAS1*, *ATRX*, *HRAS*, *TP53*, *MET*, *BRAF*, *IDH1*, *IDH2*, *SETD2*, *KDM2B*, *FGFR1*, and *FLT1* [14,19,20,21,22,23,24,25]. The presence of a mutation in specific genes is associated with an increased risk of metastasis and multifocality, as well as syndromic disorders and tumor distribution [1,7,26]. Furthermore, PPGLs have been demonstrated to exhibit clustering according to mutations in specific genes, which have been shown to indicate disparate molecular mechanisms underlying tumor pathogenesis [27].

It has been shown that HNPGLs are primarily linked to mutations in *SDHB*, *SDHC*, and *SDHD* genes, which encode subunits of the succinate dehydrogenase complex [28]. The syndromic presentation of HNPGLs is associated with mutations in *SDHD* (paraganglioma syndrome type 1, PGL1), *SDHAF2* (PGL2), *SDHC* (PGL3), *SDHB* (PGL4), *SDHA* (PGL5), *VHL* (von Hippel–Lindau syndrome), *HIF-2α* (paraganglioma–somatostatinoma–polycythemia), and *NF1* (neurofibromatosis type 1 syndrome) [29]. Patients with *SDHB*-mutated HNPGLs exhibited an elevated risk of developing metastasis, with rates up to 83% [26,30]. Additionally, metastasizing HNPGLs have been reported in association with *SDHD*, *SDHC*, *FH*, *TERT*, and *ATRX* mutations [31,32,33,34]. Ricketts et al. demonstrated that HNPGLs manifest as multiple tumors in 23% of *SDHD*-mutated cases and in 9% of *SDHB*-mutated cases [35]. Furthermore, *SDHx* variants have been linked to an elevated risk of recurrence [36]. The alteration of molecular pathways resulting from genetic mutations represents a promising avenue for the development of personalized therapies for HNPGLs [5]. Therefore, HNPGL exhibits pronounced phenotype–genotype correlations, and the identification of genetic mutations is crucial for the diagnosis of clinical features (syndromic, familial, metastasizing, and multiple tumors) and the selection of a therapeutic strategy. Nevertheless, the available genetic data for HNPGLs remains limited. Due to their rarity, HNPGLs are frequently grouped with PCCs and sympathetic extra-adrenal PGLs for studies, which does not fully elucidate the fundamental molecular genetic basis of these tumors.

The present study is an investigation of novel genetic events underlying HNPGLs. The exome data of a representative set of HNPGLs, comprising 152 tumors obtained from 140 patients, were subjected to analysis. A search for genetic variants classified as pathogenic or likely pathogenic in curated databases of genetic variants or predicted as deleterious by pathogenic prediction algorithms in genes not related to the main 12 drivers for PPGLs was conducted. The functional analysis of mutated genes revealed metabolic and signaling pathways that may be deregulated in HNPGLs. Furthermore, a gene association network was constructed. The results obtained facilitate a more comprehensive understanding of the genetic etiology of HNPGLs.

## 2. Results

### 2.1. A Spectrum of Potentially Deleterious Variants

**Figure 1:**
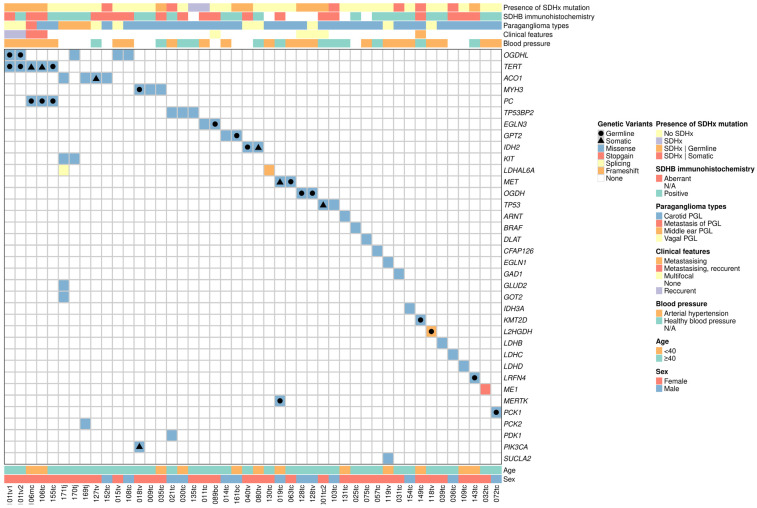
A spectrum of genetic variants identified in non-susceptibility genes across a cohort of patients with head and neck paragangliomas. The bottom of the oncoprint includes the identification number of patients with head and neck paragangliomas of different localizations (carotid, vagal, and middle ear), as well as the age and sex of patients. The top of the oncoprint plot displays the following clinical and pathological characteristics: *SDHx* mutations, SDHB immunohistochemical pattern, tumor localization, features of the clinical course, and blood pressure. The right-hand side of the plot provides a list of the mutated genes. The center section of the oncoprint illustrates the types of each variant (missense, stop-gain, splicing, and frameshift) represented by different colors. Germline or somatic status, where available, is indicated by black figures. PGL, paraganglioma. *SDHx*, genes encoding subunits of the succinate dehydrogenase complex.

A comprehensive exome analysis was conducted on 152 HNPGLs (140 patients) using the filtering strategy (see Material and Methods) to identify potentially deleterious variants in genes that are not on the list of known drivers for PPGLs (*SDHA*, *SDHB*, *SDHC*, *SDHD*, *SDHAF2*, *MAX*, *FH*, *NF1*, *RET*, *TMEM127*, *VHL*, and *SLC25A11*). A total of 36 distinct genes and 53 potentially deleterious variants have been identified in 46 tumors (43 patients, including 2 cases with analyzed recurrent or metastatic samples and 1 patient with multifocal tumors) (Supplementary Table S1). A total of twelve genes, including *ACO1*, *EGLN3*, *GPT2*, *IDH2*, *KIT*, *LDHAL6A*, *MET*, *MYH3*, *OGDHL*, *PC*, *TERT*, and *TP53BP2*, were identified as harboring more than one potentially deleterious variant within the studied set of HNPGLs (Figure 1).

A total of 94% (50/53) of the identified variants were missense mutations (Figure 1). Two cases were identified in which frameshift variants were present in the *L2HGDH* and *LDHAL6A* genes. Slicing and stop-gain variants were observed in the *LDHAL6A* and *ME1* genes, respectively. To determine the clinical significance of the identified variants, they were subjected to interpretation using the ClinVar database, in addition to the American College of Medical Genetics and Genomics (ACMG) and the Association for Molecular Pathology (AMP) 2015 (InterVar) and VarSome classifications. The majority of the identified variants were classified as variants of uncertain significance. Nevertheless, all the variants were found to possess a high pathogenic score, as predicted by a multitude of in silico algorithms, including PolyPhen-2, SIFT, CADD, DANN, LRT, MutationTaster, FATHMM, and others (Supplementary Table S2). The identified variants are situated within protein-coding regions and are expressed in the major gene transcripts. Computational methods have indicated that they have the potential to directly alter the protein sequence. The variants can lead to abnormal protein folding, protein instability, and function weakening or complete loss of function. The predicted deleterious functional effects of these variants suggest that they may be involved in tumorigenesis. Seven variants identified in the *ARNT*, *IDH2*, *L2HGDH*, *MYH3*, *PIK3CA*, and *TERT* genes were classified as pathogenic or likely pathogenic.

In the cohort of patients carrying identified potentially deleterious variants, 56% (24/43) exhibited no mutations in susceptibility genes. In 44% (19/43) of cases, pathogenic or likely pathogenic mutations in the *SDHB*, *SDHC*, and *SDHD* genes were observed. It is noteworthy that five of the sixteen *SDHx* mutations with known status were somatic. The potentially deleterious variants in non-susceptibility genes of more than half of the patients may contribute to the pathogenesis of HNPGLs. However, eight tumors lacking *SDHx* mutations were observed to display aberrant SDHB staining, indicating a disruption of the SDH complex potentially caused by alternative mechanisms, such as *SDHx* promoter methylation, chromosomal rearrangements, and so forth (Figure 1 and Supplementary Table S1) [37,38]. Therefore, it can be concluded that alterations in the SDH complex may be a primary factor in the development of these HNPGLs.

The germline or somatic mutation status was determined for 22 of the 53 identified variants. Six somatic variants were identified in the *ACO1*, *IDH2*, *MET*, *PIK3CA*, *TERT*, and *TP53* genes. A total of 16 germline potentially deleterious variants were identified in the *EGLN3*, *GPT2*, *IDH2*, *KMT2D*, *L2HGDH*, *LRFN4*, *MET*, *MERTK*, *MYH3*, *OGDH*, *OGDHL*, *PC*, *PCK1*, and *TERT* genes. Moreover, 24 identified variants with unknown mutation status exhibited variant allele frequency (VAF) values close to 0.5 (between 0.35 and 0.65, accounting for sequencing errors), suggesting a potential heterozygous state and germline status.

The frequency of potentially deleterious variants was found to be higher in carotid paragangliomas (32%, 34/106), followed by approximately equal frequencies in vagal (26.5%, 9/34) and middle ear paragangliomas (25%, 3/12). The mean age of carriers was 51 years, with a range of 28 to 68 years. The male-to-female ratio was 13:5. A total of 65% (24/37) of patients with known blood pressure levels demonstrated evidence of arterial hypertension (Supplementary Table S1).

### 2.2. Functionality of Mutated Genes

To examine the functional associations between the proteins encoded by the identified 36 genes, we utilized the STRING database. The *LDHB* gene was excluded from the analysis due to its absence from the database. The resulting protein interaction network comprised 35 nodes and 163 edges, with a protein–protein interaction enrichment value of less than 1.0 × 10^−16^ (Supplementary Figure S1). The network exhibited a high degree of protein–protein interactions, including those determined through experimentation, reported in databases, and identified through gene neighborhood, co-occurrence, co-expression, protein homology, and text mining. This suggests that these proteins are closely associated with respect to their biological roles. However, four proteins (CFAP126, MYH3, MERTK, and LRFN4) did not exhibit any interactions with other proteins within the network. Seventeen of the identified proteins are involved in mitochondrial function.

**Figure 2:**
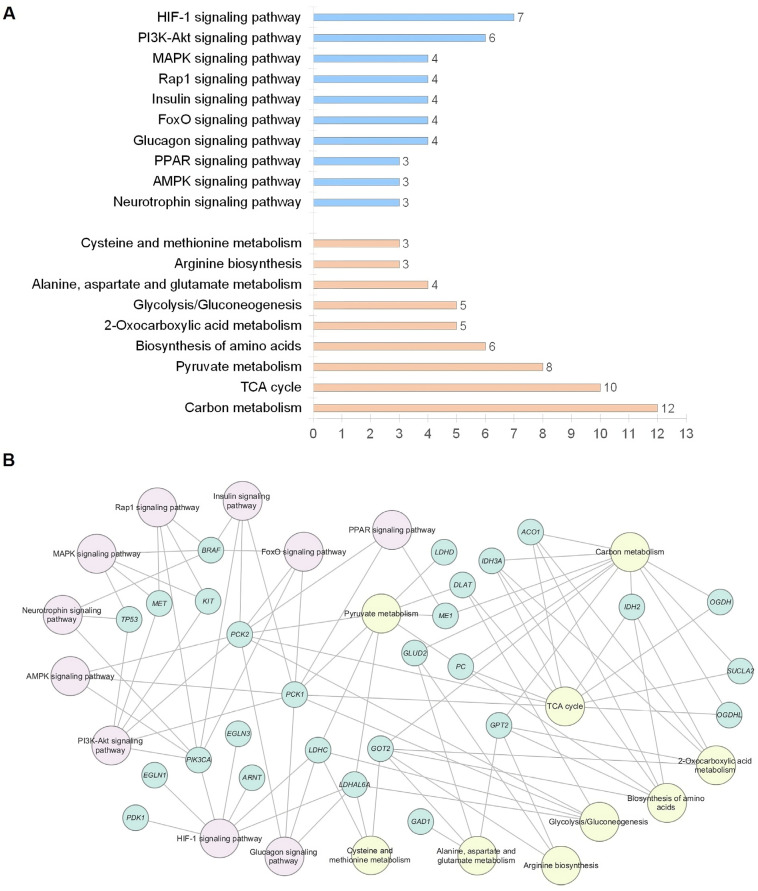
A functional enrichment analysis of mutated genes in head and neck paragangliomas based on the Kyoto Encyclopedia of Genes and Genomes (KEGG). (**A**) The number of genes categorized according to their involvement in the KEGG signaling and metabolic pathways. The blue columns indicate the number of identified mutated genes involved in signaling pathways. The orange columns indicate the number of genes involved in metabolic pathways. (**B**) The functional network showing multiple associations between mutated genes. Signaling and metabolic pathways are indicated by pink and yellow, respectively. The mutated genes are indicated by green coloring.

According to KEGG functional enrichment analysis, the majority of mutated genes are involved in metabolic pathways, such as the tricarboxylic acid (TCA) cycle, carbon metabolism, pyruvate metabolism, 2-oxocarboxylic acid metabolism, glycolysis, and gluconeogenesis. A number of genes have been identified as participating in known cancer-related signaling pathways, including HIF1, PI3K-Akt, FoxO, AMPK, MAPK, and Ras (FDR ≤ 0.01) (Figure 2).

## 3. Discussion

This study presented a range of potentially deleterious variants and a list of genes that appear to be directly associated with the pathogenesis of HNPGLs. A total of 36 genes belonging to different functional classes were identified in association with HNPGLs. The majority of genes exhibited variants of uncertain significance, whereas six genes (*ARNT*, *IDH2*, *L2HGDH*, *MYH3*, *PIK3CA*, and *TERT*) demonstrated pathogenic or likely pathogenic variants. The *TERT* gene was found to carry both germline and somatic variants. Individual cases of PPGLs with *TERT* mutations have been reported in the literature [39,40]. It has been shown that *TERT* alterations in PPGLs are frequently associated with *SDHB* germline mutations and worse prognosis [41]. The patients in our study with likely pathogenic *TERT* variants also had *SDHB* mutations. The metastatic case was previously subjected to comprehensive genetic analysis [33].

The *IDH2* gene encodes for isocitrate dehydrogenase 2, a mitochondrial enzyme that catalyzes the oxidative decarboxylation of isocitrate to 2-oxoglutarate. One of the most prevalent mutations in the *IDH2* gene is located in the active site of the enzyme at arginine 172. This mutation is associated with a variety of neoplasms, including acute myeloid leukemia, hepatocellular carcinoma, and glioblastoma [42,43,44]. It is noteworthy that the R172 mutation was identified as somatic in all cases submitted to the ClinVar database. Recent reports have documented the presence of somatic R172G *IDH2* in carotid paragangliomas [25,45]. In this study, a somatic R172K *IDH2* variant was identified in a patient with vagal paraganglioma who did not carry mutations in any susceptibility genes. *IDHx* mutations are typically exceedingly rare in PPGLs. To date, the *IDH2* mutations have only been identified at the R172 hotspot in HNPGLs [25,46].

In a patient with CPGL, we identified a pathogenic activating mutation in the hotspot H1047 region of the kinase domain in the *PIK3CA* gene. This variant has been previously identified in various cancers, predominantly in a somatic allele origin, and has been shown to result in the activation of the PI3K/AKT/mTOR pathway and oncogenic cell transformation [47,48,49]. A pathogenic somatic *PIK3CA* variant has only been reported once in the context of PPGLs, in a patient with bladder paraganglioma who also carried the H1047R mutation [50]. Furthermore, we have recently demonstrated the transcriptional activation of the PI3K-Akt signaling pathway in vagal paragangliomas [51]. In light of these findings, it can be proposed that *PIK3CA* represents a novel PPGL-associated gene, and the activation of its downstream pathways may represent one of the mechanisms responsible for the pathogenesis of PPGLs. Moreover, tumors harboring *PIK3CA* mutations (particularly H1047R) demonstrated responsiveness to anticancer therapy with inhibitors of the PI3K/AKT/mTOR pathway [52,53]. This may be a promising avenue for personalized targeted therapy in patients with PPGLs.

The *L2HGDH* gene encodes for L-2-hydroxyglutarate dehydrogenase, which oxidizes L-2-hydroxyglutarate (L-2-HG) to alpha-ketoglutarate (αKG) in mitochondria. A germline frameshift variant in the *L2HGDH* gene was identified in a patient with VPGL. Germline biallelic loss of this enzyme results in the severe neurometabolic disorder L-2-hydroxyglutaric aciduria [54]. To date, no *L2HGDH* variants have been previously identified in PPGLs. However, the gene has been included in the panel of candidate metabolic genes associated with PPGLs proposed by Gieldon et al. [55]. Furthermore, 2-HG is a competitive inhibitor of αKG, and aberrant accumulation of this metabolite can result in the inhibition of multiple αKG-dependent dioxygenases, leading to a hypermethylation phenotype that is commonly observed in tumors harboring *SDHx* and *IDHx* mutations [49,50]. Consequently, alterations in *L2HGDH* may potentially exhibit an epigenetic pattern analogous to that observed in *SDHx-/IDHx*-mutated tumors.

In a novel finding, likely pathogenic variants were also identified in the *ARNT* and *MYH3* genes. The *ARNT* gene encodes for a stably expressed HIF1β, which functions as a cofactor for transcriptional regulation by HIFs [56]. The HIF1α/HIF2α-HIF1β heterodimer complex regulates the activity of hundreds of genes involved in a variety of biological processes, including angiogenesis, glycolysis, cell proliferation, inflammation, autophagy, apoptosis, extracellular matrix remodeling, lipid metabolism, and others [56,57]. Aberrant HIFα stability resulting from mutations targeting hypoxia pathway-related genes (*SDHx*, *VHL*, *PHDs*, and *EPAS1*) is a common occurrence in PPGLs and is regarded as a mechanism of pathogenesis [58]. Therefore, alterations in *ARNT* can also be presented as an oncogenic event within the HIF network in PPGLs. Furthermore, recent studies have identified multiple additional pathways through which *ARNT* may contribute to tumor initiation, progression, and drug resistance, including the fibronectin/integrin β1/FAK, Myc/Miz/CDKN2B, NF-κB, p38α-MAPK, and canonical AHR/ARNT signaling pathways [59,60,61,62]. It is noteworthy that a likely pathogenic *ARNT* variant was identified in an early-onset patient who did not have deleterious mutations in any other genes. Moreover, this variant has the potential to be germline (VAF value close to 0.5). This suggests that the *ARNT* gene alteration may play a significant role in the pathogenesis of HNPGL in this patient.

In the *MYH3* gene, three variants were observed in different patients with CPGLs (one likely pathogenic variant and two variants of uncertain significance). All three variants appear to be germline, exhibiting a VAF of approximately 0.5. The *MYH3* gene encodes for an embryonic myosin heavy chain, which is highly expressed during prenatal development. Pathogenic mutations in this gene have been identified as the cause of Freeman–Sheldon and Sheldon–Hall syndromes [63]. They have also been associated with chromosome 17p13.3 duplication syndrome and contractures, pterygia, and spondylocarpotarsal fusion syndrome 1A. A number of studies have shown a significant correlation between *MYH3* expression and *PTEN* mutation in prostate cancer [64], tongue cancer metastasis [65], and breast cancer prognosis [66]. *MYH3* has been identified as a gene associated with disulfideptosis in colon adenocarcinoma [67]. Therefore, the available evidence suggests that *MYH3* plays a role in tumor initiation and progression, although its specific function remains to be elucidated.

In the set of HNPGLs studied, 30 out of 36 genes exhibited variants of uncertain significance that have not yet been proven to be linked with pathologies. Nevertheless, all of these variants were found to possess a high pathogenic score as predicted by in silico tools, a low population frequency, and a high positional conservation score, which together suggest the potential for them to be deleterious. Moreover, the majority of the identified variants were present in genes that had previously been reported in association with PPGLs or were suspected of being involved in the pathogenesis of PPGLs due to their participation in metabolism, epigenetic regulation, and cancer-associated pathways [55]. Our results provide evidence that alterations in these genes can indeed occur in PPGLs, particularly in HNPGLs. In addition, it provides insights into the genotype–phenotype correlation for variants of uncertain significance.

To gain further insight into the role of the identified genes and genetic variants in tumorigenesis, we presented data on the mutation status of susceptibility genes in the same tumors. The majority of patients with HNPGLs had mutations in the *SDHB*, *SDHC*, and *SDHD* genes. *SDHx* mutations result in the dysfunction of succinate dehydrogenases, leading to the accumulation of the oncometabolite succinate, the stabilization of HIFs, and DNA and histone hypermethylation [6]. In addition, we examined the available data on immunohistochemical staining of SDHB subunits, which reflect the stability of succinate dehydrogenase and serve as surrogate markers for *SDHx* gene alterations, with the aim of identifying any overlooked *SDHx* changes (Supplementary Table S1) [68]. The results indicate that the majority of mutated genes were observed in tumors negative for *SDHx* mutations (*BRAF*, *DLAT*, *CFAP126*, *EGLN1*, *GLUD2*, *GOT2*, *IDH3A*, *KIT*, *LDHAL6A*, *LDHB*, *LDHD*, *ME1*, *MERTK*, *PCK1*, *PCK2*, *SUCLA2*, *ARNT*, *L2H6DH*, and *PIK3CA*). The *ACO1*, *EGLN3*, *GPT2*, *OGDHL*, *PC*, *TP53BP2*, *IDH2*, *MYH3*, and *TERT* genes exhibited mutations in both *SDHx*-associated and apparently sporadic tumors. The analysis revealed that only seven mutated genes (*GAD1*, *LDHC*, *LRFN4*, *OGDH*, *PDK1*, and *TP53*) were exclusively identified in *SDHx*-mutated tumors. Moreover, tumors with variants of uncertain significance in *BRAF*, *CFAP126*, *EGLN1*, *GLUD2*, *GOT2*, *IDH3A*, *KIT*, *LDHAL6A*, *LDHB*, *ME1*, *PCK1*, *PCK2*, and *SUCLA2* genes or pathogenic variants in *PIK3CA* and *L2HGDH* genes did not present *SDHx* mutations or aberrant SDHB staining. This suggests that alterations in these genes may represent a driver event for HNPGLs. Previously, variants in the *BRAF*, *EGLN1*, *GOT2*, *PCK2*, and *SUCLA2* genes have also been identified in apparently sporadic PPGL cases, supporting the possibility that these genes may act as predisposition genes for PPGLs [23,24,69,70].

The functional network analysis of the identified mutated genes revealed numerous interactions between them and facilitated the visualization of the biological pathways in which they are involved. The gene network was generally enriched in metabolic and signaling pathways with a predominance of carbon metabolism, TCA cycle, pyruvate metabolism, amino acid biosynthesis, and HIF-1 and PI3K-Akt signaling pathways in which more than five genes were involved simultaneously. An excellent previous study demonstrated the clustering of PPGLs based on mRNA expression profiles into at least three subtypes (kinase signaling, pseudohypoxia, and Wnt-altered) depending on the mutational landscape [27]. Accordingly, the molecular composition of the gene network was partially expected in terms of changes in the major cellular metabolic pathways, HIF signaling, and RAS/MAPK signaling. Activation of the PI3K/Akt/mTOR pathway was previously observed in primary cultures of PPGL cells, and its blockade resulted in the inhibition of cell proliferation [71]. The inhibition of pheochromocytoma cell proliferation and tumor growth by the suppression of PI3K/Akt/mTOR has also been demonstrated in a number of in vitro studies, indicating its functional role in tumorigenesis [72,73]. The PI3K/Akt, FOXO, AMPK, MAPK, Rap1, and PPAR pathways are interconnected in a signaling network that plays an essential role in the regulation of cell proliferation and survival, angiogenesis, inflammation, and homeostasis [74,75,76,77]. It is reasonable to posit that the deregulation of any of the nodes in this network is likely to be an important factor in tumor promotion. Additionally, PPGLs are often associated with significant glycemic alterations, the underlying mechanisms of which are currently under investigation [78]. It is conceivable that genetic alterations of genes involved in the insulin and glucagon signaling pathways may represent a potential mechanism underlying impaired glucose homeostasis in PPGLs. Consequently, alterations in identified genes exert influence over multiple biological pathways, including those that are the same or related to those affected by susceptibility genes, and cancer-associated signaling pathways.

It should be noted that the study is subject to a number of limitations. The first limitation is the absence of experimental evidence demonstrating the functional impact of missense variants on RNA or protein. Nevertheless, numerous computational algorithms indicated that these variants may have a deleterious impact. Moreover, the majority of identified variants are situated within genes that are implicated in onco-associated molecular pathways, including those that have been altered in PPGLs. Some of the identified variants were present in tumors that lacked any mutations in the known driver genes. This indicates that these alterations may play a substantial role in the pathogenesis of HNPGL. A second limitation is the unavailability of paired normal tissue for numerous patients, due to the use of an archival collection of HNPGLs, given their rarity. Consequently, it was not possible to ascertain the germline/somatic mutation status for all identified genetic variants.

Future research directions may include experimental verification of the functional impact of identified variants with uncertain clinical significance. Additionally, the identified mutated genes may be tested on genetic variants in other distinct population cohorts with HNPGLs and PPGLs. The role of these gene alterations in inherited and/or sporadic tumors may also be studied, as well as their association with an aggressive disease course.

## 4. Materials and Methods

### 4.1. Patient Cohort and Specimens

A total of 152 archived formalin-fixed paraffin-embedded (FFPE) tumors and 57 normal (blood and/or lymph node) tissues obtained from 140 patients with HNPGLs were included in the study. The samples were collected at the Vishnevsky Institute of Surgery Russian Academy of Medical Sciences, and the study was approved by the ethics committee of the institute (ethics committee approval no. 007/18, 2 October 2018). Informed consent was obtained from all patients involved in the study. The research was conducted in accordance with the Declaration of Helsinki (1964). The clinical and pathological characteristics of the patients are presented in Table 1.

### 4.2. DNA Extraction

The DNA was extracted from the FFPE tissues using the High Pure FFPET DNA Isolation Kit (Roche, Basel, Switzerland) according to the manufacturer’s instructions. The DNA was isolated from blood using the MagNA Pure Compact Nucleic Acid Isolation Kit I (Roche, Basel, Switzerland) on a MagNA Pure Compact instrument (Roche, Basel, Switzerland). The amount of DNA was determined using a Qubit 2.0 fluorometer (Thermo Fisher Scientific, Waltham, MA, USA). The quality of the DNA extracted from FFPE samples was evaluated using an Agilent 2100 Bioanalyzer (Agilent Technologies, Santa Clara, CA, USA). The DNA samples were stored at a temperature of −32 °C.

### 4.3. Exome Library Preparation and Sequencing

Exome libraries were prepared using the Nextera Rapid Capture Exome Kit and TruSeq Exome Library Prep Kit (Illumina, San Diego, CA, USA) in accordance with the manufacturer’s instructions, with the exception of the mechanical/enzymatic DNA fragmentation step, which was adapted to account for the quality of the DNA. The quality of the prepared libraries was evaluated using an Agilent 2100 Bioanalyzer (Agilent Technologies, Santa Clara, CA, USA). The quantity of the prepared libraries was determined using a Qubit 2.0 fluorimeter (Thermo Fisher Scientific, Waltham, MA, USA). High-throughput sequencing was conducted in the paired-end mode (76 × 2 bp) on a NextSeq 500 System (Illumina, San Diego, CA, USA) with an estimated average coverage of at least 300×. The sequencing data are accessible via the NCBI Sequence Read Archive, BioProject PRJNA755885.

### 4.4. Bioinformatics Analysis

The raw sequencing reads were subjected to quality control using FASTQC (v0.11.9, https://www.bioinformatics.babraham.ac.uk/projects/fastqc/, accessed on 20 June 2024) and were trimmed for the removal of adapter sequences, the trimming of low-quality base pairs, and the filtering out of low-quality reads (Q < 30) using Trimmomatic (v0.39) [79]. The trimmed reads were then aligned to the reference human genome (GRCh37/hg19) using BWA-MEM (v0.7.17) [80] with further processing of the BAM files conducted using Samtools (v1.10) [81] and Picard-tools (v2.21.3, http://broadinstitute.github.io/picard/, accessed on 20 June 2024). Four distinct tools, HaplotypeCaller GATK (v4.1.2) [82], Freebayes (v1.3.5) [83], Strelka2 (v2.9.10) [84], and VarDict (v1.8.2) [85], were utilized for variant calling, thereby ensuring the reliable detection of variants at specific loci.

The identified genetic variants were subsequently annotated using ANNOVAR [86]. The population frequency of the variants was estimated using the gnomAD, 1000 Genomes, ExAC, and Kaviar databases. The following in silico algorithms were used to predict the pathogenicity of the identified variants: Polyphen2 (HDIV and HVAR), SIFT, SIFT4G, VEST4, CADD, DANN, LRT, MutationTaster, MutationAssessor, FATHMM (MKL and XF), PROVEAN, Meta (SVM and LR), M-CAP, REVEL, MutPred, MVP, DEOGEN2, BayesDel (addAF and noAF), ClinPred, and LIST-S2. The degree of conservation at the variant position was evaluated using the phastCons and PhyloP tools. The following criteria were employed for filtering of potentially deleterious variants: (1) average coverage ≥ 20, (2) variant detection by at least three callers, (3) variant type as nonsynonymous missense, reading frameshift, loss/gain of stop codon/start codon, or deletion/insertion, (4) variant occurrence in the main gene transcripts, (5) prediction as deleterious by majority of in silico prediction algorithms, (6) low population frequency (<1%) to exclude gene polymorphisms, and (7) classification as variants of uncertain significance (US), probably pathogenic (LP), or pathogenic (P) according to ClinVar, ACMG/AMP 2015 (InterVar), and VarSome. A variant was considered to be germline if it was identified in normal tissue obtained from the same patient. The lymph node tissues were subjected to histological verification, and the absence of tumor cells (metastasis) was confirmed. The STRING database (v12.0) was used for the analysis of functional protein–protein association networks with the default parameters [87]. The Cytoscape software (v3.10.2) was employed for the visualization of gene functional associations [88].

## 5. Conclusions

The presence of multiple variants with pathogenic or likely pathogenic clinical significance or undetermined pathogenicity (but with a high predisposition to be pathogenic) in numerous genes indicates high heterogeneity of HNPGLs and different potential molecular pathways for their pathogenesis.

Although the occurrence of mutations in the *TERT*, *IDH2*, and *PIK3CA* genes is uncommon among HNPGLs, they should be included in the list of somatically mutated susceptibility genes for these tumors. Moreover, the identification of specific hotspots in *IDH2* (R172) and *PIK3CA* (H1047) in PPGLs (based on our own data and previously published findings) is of importance for diagnostic purposes. The *L2HGDH*, *ARNT*, and *MYH3* genes, which have been identified as carrying likely pathogenic or pathogenic variants, may be considered novel genes responsible for HNPGL pathogenesis on a preliminary basis.

## Figures and Tables

**Table 1:** The clinical and pathological characteristics of the patient cohort.

Characteristic	Number of Patients, n
Total patients	140
Total tumors	152
Sex	
Male	24
Female	116
Age at diagnosis	
≤40	45
>40	95
Mean	48.9
Tumor localization	
Carotid paragangliomas	106
Vagal paragangliomas	34
Middle ear paragangliomas	12
Clinical feature	
Multifocal	12
Recurrent	8
Metastasis	3

## Data Availability

All data generated or analyzed during this study are included in this published article. The exome sequencing data are available in the NCBI SRA under the accession number PRJNA755885.

## Supplementary Materials

The following supporting information can be downloaded at: https://www.mdpi.com/article/10.3390/ijms252312762/s1.

## Abbreviations

HNPGLs: head and neck paragangliomas; PGLs, paragangliomas; PCCs, pheochromocytomas; PPGLs, paragangliomas and pheochromocytomas; CPGLs, carotid paragangliomas; VPGLs, vagal paragangliomas; MEPGLs, middle ear paragangliomas; PGL1–5, paraganglioma syndrome type 1–5; *SDHx*, four genes (*SDHA*, *SDHB*, *SDHC*, and *SDHD*) encoding subunits of the succinate dehydrogenase complex; ACMG/AMP, The American College of Medical Genetics and Genomics and the Association for Molecular Pathology; VAF, variant allele frequency; TCA cycle, tricarboxylic acid cycle; KEGG, the Kyoto Encyclopedia of Genes and Genomes; FFPE, formalin-fixed paraffin-embedded.
