# Supplementary material for: Non-Susceptibility Gene Variants in Head and Neck Paragangliomas
Source: Int J Mol Sci. 2024 Nov 27;25(23):12762. doi: 10.3390/ijms252312762 (PMC11641674; doi:10.3390/ijms252312762)
Supplement: Supplementary file 1 [file ijms-25-12762-s001.zip › Supplementary Table S1 and Figure S1.pdf]

Supplementary Table S1. **Potentially deleterious variants identified in candidate genes in HNPGLs and association with clinical-pathological characteristics of the patients.**

Gene	Protein	Variant type	Variant description	Status	VAF	Maximum population frequency	Tumor Id	ClinVar	ACMG/AMP 2015 (InterVar)	VarSome	Variants in susceptibility genes	Tumor localization	Sex	Age	Clinical features	Arterial hypertension	IHC SDHB
ACO1	Aconitase 1	Missense	NM_002197: c.A622G, p.T208A (chr9: 32418473, rs61753543)	Somatic	0.44	0.005	127tv	NA	US	US	No	Vagal	F	49	No	No	-
				NA	0.68		169tj				No	Middle ear	F	50	NA	NA	+
				NA	0.44		171tj				No	Middle ear	F	57	No	NA	+
		Missense	NM_002197: c.T1708G, p.F570V (chr9: 32430554, rs769809176)	NA	0.68	0	152tc	NA	US	US	*SDHC* somatic	Carotid	M	63	No	NA	*
ARNT	Aryl Hydrocarbon Receptor Nuclear Translocator	Missense	NM_001668: c.A868G, p.R290G (chr1: 150804811, rs763732376)	NA	0.53	0.00001	131tc	NA	US	LP	No	Carotid	F	28	No	No	NA
BRAF	B-Raf Proto-Oncogene, Serine/Threonine Kinase	Missense	NM_004333: c.G533A, p.R178Q (chr7: 140508767,	NA	0.39	0.00006	025tc	US	US	US	No	Carotid	F	52	No	NA	+

			rs746348396)														
DLAT	Dihydrolipoamide S-acetyltransferase	Missense	NM_001931: c.C1114T, p.L372F (chr11: 111910106, rs782124389)	NA	0.27	6.5E-06	075tc	NA	US	US	No	Carotid	F	61	No	Yes	NA
CFAP126	Cilia And Flagella Associated Protein 126	Missense	NM_001013625: c.C155T, p.S52F (chr1: 161336004)	NA	0.25	0	057tc	NA	US	US	No	Carotid	F	42	No	No	+
EGLN1	Egl-9 Family Hypoxia Inducible Factor 1	Missense	NM_022051: c.A994G, p.K332E (chr1: 231509743)	NA	0.48	0	119tv	NA	US	US	No	Vagal	F	38	Craniomeningioma	Yes	+
EGLN3	Egl-9 Family Hypoxia Inducible Factor 3	Missense	NM_022073: c.G421A, p.G141S (chr14: 34400358, rs759224695)	NA	0.47	0.0001	011tc	NA	US	US	*SDHB*	Carotid	F	70	No	Yes	-
		Missense	NM_022073: c.A515T, p.K172I (chr14: 34398381)	Germline	0.54	0	089bc	NA	US	US	No	Carotid	F	42	Multifocal	NA	-

GAD1	Glutamate Decarboxylase 1	Missense	NM_000817: c.C623T, p.T208M (chr1: 171693378, rs754891860)	NA	0.425	0.0008	031tc	NA	US	US	*SDHB* somatic	Carotid	F	58	No	Yes	+
GLUD2	Glutamate Dehydrogenase 2	Missense	NM_012084: c.A715G, p.T239A (chrX: 120182253)	NA	0.47	0	171tj	NA	US	US	No	Middle ear	F	57	No	NA	+
GOT2	Glutamic-Oxaloacetic Transaminase 2	Missense	NM_002080: c.C233T, p.P78L (chr16: 58757663, rs765537389)	NA	0.51	0.0004	171tj	US	US	US	No	Middle ear	F	57	No	NA	+
GPT2	Glutamic--Pyruvic Transaminase 2	Missense	NM_133443: c.C634T, p.P212S (chr16: 46943653)	NA	0.73	0	014tc	NA	US	US	No	Carotid	M	68	No	Yes	+
		Missense	NM_133443: c.G971A, p.G324E (chr16: 46952603, rs201383826)	Germline	0.36	0.003	161bc	NA	US	US	*SDHD* germline	Carotid	M	38	No	NA	+

IDH2	Isocitrate Dehydrogenase (NADP(+)) 2	Missense	NM_002168: c.T1154C, p.L385P (chr15: 90628257)	Germline	0.47	0	040tv	NA	US	US	*SDHB* germline	Vagal	F	56	No	NA	-
		Missense	NM_002168: c.G515A, p.R172K (chr15: 90631838, rs121913503)	Somatic	0.33	6.5E-06	080tv	P/LP	LP	P	No	Vagal	F	39	No	No	+
IDH3A	Isocitrate Dehydrogenase (NAD(+)) 3 Catalytic Subunit Alpha	Missense	NM_005530: c.G893A, p.G298D (chr15: 78458520, rs368666636)	NA	0.53	7.7E-05	154tc	US	NA	US	No	Carotid	M	60	Prostate adenoma	Yes	+
KIT	KIT Proto-Oncogene, Receptor Tyrosine Kinase	Missense	NM_000222: c.G793A, p.G265S (chr4: 55569926, rs1060502570)	NA	0.5	0.0002	170tj	US	US	US	No	Middle ear	F	67	No	NA	+
		Missense	NM_000222: c.G2926A, p.V976I (chr4: 55604718, rs773955363)	NA	0.395	0.00001	171tj	US	US	US	No	Middle ear	F	57	No	NA	+

KMT2D	Lysine Methyltransferase 2D	Missense	NM_003482: c.A7687C, p.N2563H (chr12: 49433866)	Germline	0.54	0	149tc	NA	US	US	*SDHD* somatic	Carotid	F	19	Metastasis	No	*
L2HGDH	L-2-Hydroxyglutarate Dehydrogenase	Frameshift	NM_024884: c.853delT, p.Y285Tfs*4 (chr14: 50735933, rs748910382)	Germline	0.41	0.0003	118tv	P	NA	P	No	Vagal	F	56	No	Yes	+
LDHAL6A	Lactate Dehydrogenase A Like 6A	Frameshift	NM_144972: c.605delG, p.S202Mfs*3 (chr11: 18497942)	NA	0.73	0	130tc	NA	US	US	No	Carotid	F	44	No	Yes	NA
		Splicing	NM_144972: c.592+1G>A (chr11: 18497187, rs145833201)	NA	0.46	0.004	171tj	NA	NA	US	No	Middle ear	F	57	No	NA	+
LDHB	Lactate Dehydrogenase B	Missense	NM_002300: c.G299A, p.R100H (chr12: 21796991, rs777019739)	NA	0.76	0.00002	039tc	NA	US	US	No	Carotid	F	68	No	Yes	+

LDHC	Lactate Dehydrogenase C	Missense	NM_002301: c.T841G, p.Y281D (chr11: 18472516)	NA	0.62	0	036tc	NA	US	US	*SDHD* somatic	Carotid	M	59	No	NA	*
LDHD	Lactate dehydrogenase D	Missense	NM_153486: c.T548C, p.M183T (chr16: 75148505)	NA	0.57	0	109tc	NA	US	US	No	Carotid	F	39	No	NA	*
LRFN4	Leucine Rich Repeat And Fibronectin Type III Domain Containing 4	Missense	NM_024036: c.C739T, p.R247W (chr11: 66625954)	Germline	0.36	0	143tc	NA	US	US	*SDHD* germline	Carotid	F	38	No	No	+
ME1	Malic Enzyme 1	Stopgain	NM_002395: c.G679T, p.E227X (chr6: 84025054)	NA	0.42	0	032tc	NA	US	US	No	Carotid	F	62	No	Yes	+
MET	MET Proto-Oncogene, Receptor Tyrosine Kinase	Missense	NM_000245:c.G1789T, p.D597Y (chr7: 116395496)	Somatic	0.35	0	019tc	NA	US	US	No	Carotid	M	35	No	No	*
		Missense	NM_000245: c.T682C, p.F228L (chr7: 116339820,	Germline	0.43	0.0002	063tc	US	US	US	No	Carotid	F	63	No	Yes	NA

			rs921083171)														
MERTK	MER Proto-Oncogene, Tyrosine Kinase	Missense	NM_006343: c.G2467C, p.E823Q (chr2: 112779952, rs55924349)	Germline	0.47	0.003	019 tc	US	US	US	No	Carotid	M	35	No	No	*
MYH3	Myosin Heavy Chain 3	Missense	NM_002470: c.T791C, p.I264T (chr17: 10550688, rs763347751)	NA	0.5	0.0007	035 tc	US	US	LP	*SDHD* germline, *RET*	Carotid	F	30	No	No	-
		Missense	NM_002470: c.C1325T, p.T442I (chr17: 10547753, rs769788909)	NA	0.48	0.0008	009 tc	US	US	US	No	Carotid	F	51	NA	NA	+
		Missense	NM_002470: c.A2392G, p.M798V (chr17: 10543684)	Germline	0.52	0	018 tv	US	US	US	No	Carotid	F	64	No	NA	+
OGDH	Oxoglutarate Dehydrogenase	Missense	NM_002541: c.A1483C, p.S495R (chr7: 44733571, rs2230446)	Germline	0.2	0.0025	128 tc	US	US	US	*SDHD* germline	Carotid	M	54	Multifocal	Yes	NA
					0.73		128 tv					Vagal					

OGDHL	2-oxoglutarate Dehydrogenase-Like	Missense	NM_018245: c.G660C, p.W220C (chr10: 50959962, rs138462834)	NA	0.52	0.002	170tj	NA	US	US	No	Middle ear	F	67	No	NA	+
		Missense	NM_018245: c.A541T, p.I181F (chr10: 50960232, rs201458076)	NA	0.4	0.0009	015tv	NA	US	US	No	Vagal	F	52	No	Yes	*
		Missense	NM_018245: c.G587C, p.R196P (chr10: 50960186)	Germline	0.46	0	101tv1	NA	US	US	*SDHB* germline	Vagal	F	46	Reccurence	Yes	-
					0.46		101tv2					Vagal (reccurence)					+
		Missense	NM_018245: c.T542C, p.I181T (chr10: 50960231, rs147143536)	NA	0.78	0.0011	108tc	NA	US	US	No	Carotid	M	48	No	Yes	-
PC	Pyruvate Carboxylase	Missense	NM_000920: c.G1003C, p.V335L (chr11: 66636336, rs768230282)	Germline	0.42	0.00001	106tc	NA	US	US	*SDHB* germline	Carotid	F	38	Metastasis, reccurence	Yes	*
					0.61		106mc					Metastasis					*

		Missense	NM_000920: c.C875A, p.T292N (chr11: 66637801, rs746397600)	Germline	0.47	6E-06	155tc	NA	US	US	No	Carotid	F	58	No	Yes	+
PCK1	PhosphoeNolpyruvate Carboxykinase 1	Missense	NM_002591: c.C1211T, p.S404L (chr20: 56139562, rs149472972)	Germline	0.55	0.003	072tc	US	US	US	No	Carotid	M	61	No	Yes	+
PCK2	PhosphoeNolpyruvate Carboxykinase 2	Missense	NM_004563: c.C349T, p.R117W (chr14: 24567485, rs778125092)	NA	0.37	0.0001	169tj	NA	US	US	No	Middle ear	F	50	No	NA	+
PDK1	Pyruvate Dehydrogenase Kinase 1	Missense	NM_002610: c.T226A, p.F76I (chr2: 173423465)	NA	0.16	0	021tc	NA	US	US	*SDHD* somatic	Carotid	M	63	No	Yes	+
PIK3CA	PhosphatidyliNositol-4,5-Bisphosphate 3-Kinase Catalytic Subunit Alpha	Missense	NM_006218: c.A3140G, p.H1047R (chr3: 178952085, rs121913279)	Somatic	0.27	6E-06	018tv	P	P	P	No	Carotid	F	64	No	NA	+

SUCLA2	Succinate-CoA Ligase ADP-Forming Subunit Beta	Missence	NM_003850: c.T484A, p.Y162N (chr13: 48562726)	NA	0.2	0	119tv	NA	US	US	No	Vagal	F	38	Craniomeningioma	Yes	+
TERT	Telomerase Reverse Transcriptase	Missence	NM_198253: c.G355T, p.V119L (chr5: 1294646)	Germline	0.61	0	101tv1	NA	US	LP	*SDHB* germline	Vagal	F	46	Reccurence	Yes	-
					0.55		101tv2					Vagal (reccurence)					+
		Missence	NG_055467.1: g.G586A (chr5: 1295228)	Somatic	0.34	NA	106tc	LP	NA	NA	*SDHB* germline	Carotid	F	38	Metastasis, reccurence	Yes	*
					0.11		106mc					Metastasis					*
		Missence	NM_198253: c.A2158G, p.I720V (chr5: 1278884, rs373072850)	Germline	0.46	0.001	155tc	NA	US	US	No	Carotid	F	58	No	Yes	+
TP53	Tumor Protein P53	Missence	NM_000546: c.A170G, p.D57G (chr17: 7579517)	Somatic	0.26	0	001tc2	US	US	US	*SDHD* germline	Carotid	F	58	Multifocal	No	*
				NA	0.62		103tc				*SDHD*	Carotid	M	57	No	No	-
TP53BP2	Tumor Protein P53 Binding Protein 2	Missence	NM_005426: c.C2932T6 p.R978C (chr1: 223971861,	NA	0.43	0.001	030tc	US	US	US	No	Carotid	M	40	No	No	-

			rs199572474)														
		Missense	NM_001031685: c.C247T, p.R83C (chr1: 224001984, rs200028775)	NA	0.39	0.0004	135tc	NA	US	US	*SDHD*	Carotid	F	50	No	No	NA
		Missense	NM_005426: c.G2680T, p.A894S (chr1: 223976806)	NA	0.53	0	021tc	NA	US	US	*SDHD* somatic	Carotid	M	63	No	Yes	+

IHC, immunohistochemistry. SDHB+, positive IHC staining. SDHB-, negative IHC staining. SDHB*, weak diffuse IHC staining. NA, data unavailable. VAF, variant allele frequency.

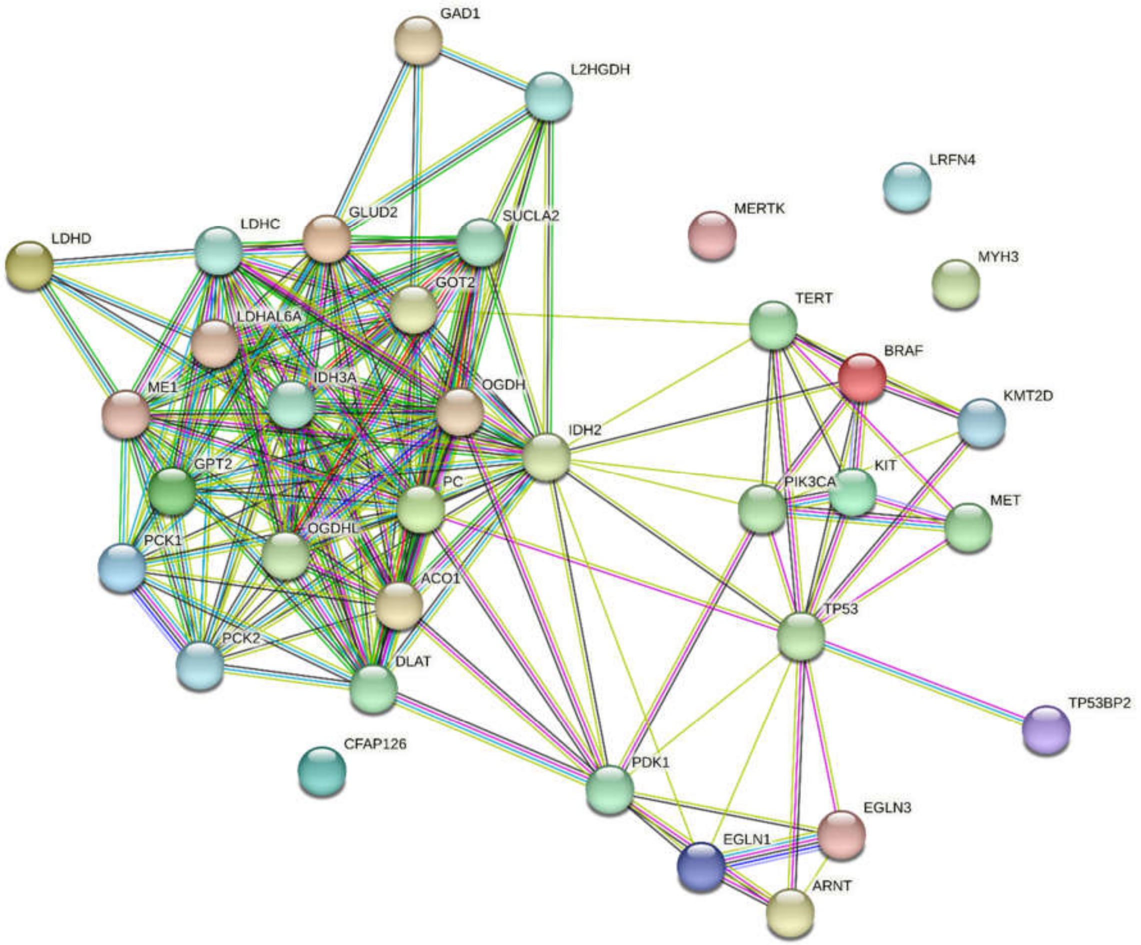

Supplementary Figure S1. **Protein-protein interaction network made with STRING database for candidate genes in HNPGLs.** Network nodes represent proteins that are coded by candidate genes. Edges represent protein-protein interactions (PPI), which are marked with the following colors: purple represents PPI that have been determined through experimental methods, turquoise represents PPI that have been sourced from curated databases, light green represents PPI that have been identified through text mining, black represents gene co-expression, light blue represents PPI that have been identified through protein homology, green represents PPI that have been identified through gene neighborhood analysis, and blue represents gene co-occurrence.
